# The transcriptional regulation of a putative hemicellulose gene, PtrPARVUS2 in poplar

**DOI:** 10.1038/s41598-024-63408-x

**Published:** 2024-06-01

**Authors:** Dan Wang, Heather D. Coleman

**Affiliations:** Department of Biology, Syracuse University, Syracuse, NY 13244 USA

**Keywords:** Poplar, Secondary cell wall, Hemicellulose biosynthesis, Transcription factors, Plant biotechnology, Plant molecular biology

## Abstract

The plant cell wall serves as a critical interface between the plant and its environment, offering protection against various stresses and contributing to biomass production. Hemicellulose is one of the major components of the cell wall, and understanding the transcriptional regulation of its production is essential to fully understanding cell wall formation. This study explores the regulatory mechanisms underlying one of the genes involved in hemicellulose biosynthesis, Ptr*PARVUS2*. Six transcription factors (TFs) were identified from a xylem-biased library to negatively regulate Ptr*PARVUS2* expression. These TFs, belonging to diverse TF families, were confirmed to bind to specific cis-elements in the PtrPARVUS2 promoter region, as validated by Yeast One-Hybrid (Y1H) assays, transient expression analysis, and Chromatin Immunoprecipitation sequencing (ChIP-seq) assays. Furthermore, motif analysis identified putative cis-regulatory elements bound by these TFs, shedding light on the transcriptional regulation of SCW biosynthesis genes. Notably, several TFs targeted genes encoding uridine diphosphate glycosyltransferases (UGTs), crucial enzymes involved in hemicellulose glycosylation. Phylogenetic analysis of UGTs regulated by these TFs highlighted their diverse roles in modulating hemicellulose synthesis. Overall, this study identifies a set of TFs that regulate *PARVUS2* in poplar, providing insights into the intricate coordination of TFs and Ptr*PARVUS2* in SCW formation. Understanding these regulatory mechanisms enhances our ability to engineer plant biomass for tailored applications, including biofuel production and bioproduct development.

## Introduction

The plant cell wall is a key structure in mitigating the interaction between the plant and its environment. The physical structure of the plant cell wall reduces insect predation, plays important roles in intercellular communication, and even prevents unwanted molecules from entering the cell^1–4^. At the same time, the cell wall accounts for the majority of the organic carbon in the world, making it important from the perspective of biomass for fuels and other products^5^.

The secondary cell wall (SCW), located between the plasma membrane and the primary cell wall, is one of the factors that allows trees to grow to such great heights^6^. The SCW is predominantly comprised of cellulose, hemicelluloses, and lignin. Over the past 50 years, much research has been carried out to elucidate the biosynthetic pathways involved in the production of cellulose and lignin^7,8^. However, until more recently hemicellulose has not been as well studied. Hemicellulose plays an important role in plant biomass^9^ and increasing or altering the content of hemicellulose has been identified as a potential way to raise the value of plant biomass for biofuels and other bioproducts.

In most vascular plants, such as poplar and *Arabidopsis*, the primary hemicellulose in the SCW is glucuronoxylan (xylan), with glucomannan and galactoglucomannan making up small proportions^10^. While cellulose is synthesized at the plasma membrane via large protein complexes, glucuronoxylan, a polymer of β-(1,4)-linked xylose with various side chains, is produced in the Golgi apparatus and transferred to the plasma membrane^11,12^.

In Arabidopsis, the IRX8 and PARVUS genes are vital for synthesizing the tetrasaccharide end sequence putatively involved in xylan biosynthesis^13^. This reducing end sequence (RES) is composed of D-Xylose, D-Glucuronic Acids, L-Rhamnose, D-Galactose, and L-Arabinose (furanose)^12^. While IRX8 operates in the Golgi, PARVUS, a member of the GT8 family, functions in the endoplasmic reticulum (ER), initiating an enzymatic stage preceding IRX8^13^. The different subcellular localizations indicate that IRX8 and PARVUS perform distinct functions in xylan biosynthesis. In Eucalyptus, expression of the PtrPARVUS2 ortholog is reduced in tension wood, indicating a putative role in stem formation^14^. In *Populus trichocarpa*, PtrPARVUS2 (Potri.002G132900) exhibits a high degree of stem tissue specificity, with heightened expression in the 7th–8th stem internodes, while its transcript could not be detected in 1st–4th stem internodes. This indicates that PtrPARVUS2 may have a crucial function in stem development^15^. Altering the expression levels of the GT8 family genes can affect xylan and lignin content, underlining their significance in SCW development^15^. Elucidating the upstream transcriptional regulation network of Ptr*PARVUS2* may offer opportunities for targeted cell wall modifications, enhancing plant biomass for tailored applications.

In *Arabidopsis*, a network of transcription factors (TFs) regulates SCW formation by coordinating the synthesis of cellulose, hemicellulose, and lignin^16^. Multiple regulatory layers, including TFs and phytohormones, contribute to cell wall regulatory control in *Arabidopsis*. However, the specific mechanisms in other vascular plants, such as trees, have yet to be elucidated. Poplar contains many duplicate genes relative to Arabidopsis and so determination of the regulatory network that controls this developmental process in poplar is key to understanding wood formation^17,18^.

In the current study, a xylem-biased library containing 42 TFs was screened by Y1H to identify the ability to bind to the Ptr*PARVUS2* promoter in vivo. We identified six TFs with the ability to regulate Ptr*PARVUS2* expression by targeting specific *cis-elements* in the promoter region. Transient expression analysis and RT-qPCR were used to determine whether these six TFs are positive or negative regulators of PARVUS2. Finally, Chromatin Immunoprecipitation sequencing (ChIP-seq) was used to identify the putative *cis-elements in planta*.

## Materials and methods

### Generation of plasmid constructs

The 2100 bp region upstream of the start codon of *PtrPARVUS2* was synthesized (Genewiz, NJ, USA). The unique restriction sites (*XbaI* and *SbfI*) were introduced to 5’ and 3’ of the promoter of Ptr*PARVUS2* (PtrPARVUS2^pro^) using the following primers: PARVUS-Fwd-XbaI and PARVUS-Rvs-SbfI (Supplemental Table S1). PtrPARVUS2^pro^ was cloned into the pGEM-T Easy vector (Promega, USA). The ligated vector was confirmed by PCR and Sanger sequencing. Using the pGFPGUSPlus binary expression vector^19^ that harbors *EGFP* gene driven by the Cauliflower Mosaic Virus 35S (CaMV35S) promoter (RRID: Addgene_64401), we replaced the CaMV35S promoter with the PtrPARVUS2^pro^ by digesting both with *XbaI* and *SbfI* followed by T4 ligation (Promega, USA). The pGFPGUSPlus-PtrPARVUS2^pro^::EGFP vector was confirmed by PCR and Sanger sequencing and transformed into *Agrobacterium tumefaciens* (GV3101).

Yeast One-Hybrid (Y1H) destination vectors pMW#2 and pMW#3 are gifts from Marian Walhout (Addgene plasmid # 13,349; http://n2t.net/addgene:13349; RRID: Addgene_13349)^20^. pGEM- T Easy-PtrPARVUS2^pro^, pMW#2 and pMW#3 were digested with *SphI* & *SalI* and *EagI* & *XbaI* allowing PtrPARVUS2^pro^ to be inserted in the upstream of *HIS3* and *LacZ* report genes through T4 ligation (Promega, USA) for Y1H assays. The pMW#2-PtrPARVUS2^pro^ and pMW#3-PtrPARVUS2^pro^ vectors were confirmed by PCR and Sanger sequencing.

The CDS sequences without stop codon of six transcription factors (Ptr*C2H2ZF1* (Potri.010G209400), Ptr*C2H2ZF2* (Potri.014G066200), Ptr*ARF5a* (Potri.002G024700), Ptr*BLH* (Potri.010G197300), Ptr*NAC127* (Potri.018G068700) and Ptr*CORONA* (Potri.003G050100)) were cloned from Y1H library. *XbaI* and *AscI* restriction sites were introduced to 5’ and 3’ of CDS sequences of Ptr*C2H2ZF1*, Ptr*C2H2ZF2* and Ptr*NAC127*, *SpeI* and *AscI* for Ptr*ARF5a*, Ptr*BLH* and Ptr*CORONA* (Supplemental Table S1). And pMDC84 binary vector, *GFP* gene driven by the Cauliflower Mosaic Virus 35S (CaMV35S) promoter was a gift from Kent Chapman (Addgene plasmid # 96,979; http://n2t.net/addgene:96979; RRID: Addgene_96979)^21^. CDS sequences and pMDC84 were digested with *XbaI* & *AscI* or *SpeI* & *AscI* allowing TF CDS sequences to be inserted between CaMV35S and GFP report genes via T4 ligation (Promega, USA). Then, pMDC84-TFs::GFP vectors were confirmed by Sanger sequencing and transformed into *Agrobacterium tumefaciens* stain (GV3101). The CDS sequences of the six transcription factors were cloned and inserted downstream of CaMV35S promoter in pGFPGUSPlus, CaMV35S::TF vectors were confirmed by PCR and Sanger sequencing and transformed into *Agrobacterium tumefaciens* (GV3101). All plasmids are listed in Supplemental Table S2.

### Plant materials

*Populus tremula* × *alba* (717-1B4; INRA France) was cultured in Woody Plant Medium (WPM) at 25 °C under a cycle of 16-h light (50 μmol m^−2^ s^−1^)/8-h dark^22^. Transgenic hybrid poplar carrying the PtrPARVUS2^pro^::EGFP were generated through Agrobacterium tumefaciens-mediated transformation as previously described^23^. Following selection on media with antibiotics, PCR and SDS-PAGE were used to confirm transgenic lines. The transgenic plants were maintained in WPM for further analysis. Experiments on plants/plant parts must confirm that the use of plants in the present study complies with international, national, and/or institutional guidelines.

### Yeast one-hybrid assays

A xylem-biased cDNA library^24^ was used for Y1H assays, which were conducted according to the protocol from Walhout Lab^20^. pMW#2-PtrPARVUS2^pro^ and pMW#3-PtrPARVUS2^pro^ were used as the destination *HIS3* bait vector and the destination *LacZ* vector. Generally, linearized destination bait vectors were co-transformed into YM4271 yeast strain (MATa, *ura3-52, his3-200, ade2-101, ade5, lys2-801, leu2-3, 112, trp1-901, tyr1-501, gal4D, gal8D, ade5::hisG*) (Clontech, USA) and plated on Synthetic Complete Supplement Mixture (SC) medium without Histidine and Uracil (–His/–Ura). The positive clones were confirmed by yeast colony PCR. Prey vectors were transformed into positive clones and plated on SC medium without Histidine, Uracil and Tryptophan (–His/–Ura/–Trp). 0 mM (control) and 80 mM 3-amino-1,2,4-triazole (3-AT) were used for *HIS3* assay, 0.012% X-Gal per plate was used for ß-Gal assay. Empty pDEST-AD vector was used as the negative control, the positive yeast stain was a gift from Sarah Hall (Syracuse University).

### Subcellular localization in onion epidermal cells

The six TF-GFP combined vectors pMDC84-CaMV35S::TF-GFP, along with CaMV35S::GFP as the positive control, were separately transferred into onion epidermal cells by *Agrobacterium*^25^. NucBlue Live Cell Strain ReadyProbes was used for DAPI staining (Thermo Fisher, USA). The fluorescence signal of GFP and DAPI was detected by fluorescence microscopy system (AxioVert.A1 inverted fluorescence microscope, Zeiss, Germany).

### Transient expression assays

The pMDC84-CaMV35S::TF-GFP and positive control 35S::GFP binary vectors were transformed into *Agrobacterium tumefaciens* strain GV3101 as previously described^26^. The whole plants of *Populus tremula* × *alba* (717-1B4; INRA France) from WPM were soaked in the *Agrobacterium* solution containing 0.015% Silwet L-77 (Lehle Seeds, USA) and then *Agrobacterium* infiltration was performed by applying vacuum three times for three minutes. The vacuumed poplar was subsequently put on paper towels to remove excess infiltration medium and moved back to cubes at 25 °C under a cycle of 16 h light and 8 h dark for 7 days. RT-qPCR and western blot were applied for detecting TF::GFP fusion protein expression. The CaMV35S::TF and negative control empty binary vectors were transformed into *Agrobacterium tumefaciens* strain GV3101, the same agroinfiltration method was applied in PtrPARVUS2^pro^::EGFP stable transgenics.

### RNA isolation and quantitative real-time PCR analysis

The tissue culture leaves were harvested after transient transformation and stored at − 80 °C until used. The tissues were ground into fine powder under liquid nitrogen. The total RNA was extracted by Tri-Xtract following manufacturer’s instruction (G-Biosciences, USA), and 1 ug RNA treated with RQ1 RNase-Free DNase (Promega, WI, USA). DNase-treated RNA was reverse-transcribed to cDNA by High-Capacity cDNA Reverse Transcription Kits (Applied Biosystems, MA, USA). The expression of *GFP* was measured using qPCR. Samples were run in triplicate using SYBR^®^ Green master mix (BIO-RAD, CA, USA) on a CFX Connect™ Real-Time PCR Detection System (BIO-RAD, CA, USA). Poplar *UBIQUITIN 11* (*UBQ11*, Potri.017G036800) and *Elongation Factor 1-β 2* (*EF1β*, Potri.015G094200) were used as reference genes^27^. The following primer sets were used for qPCR: *UBQ11*-Fwd, *UBQ11*-Rvs, *EF1β*-Fwd, *EF1β*-Rvs, *GFP*-Fwd, *GFP*-Rvs (Supplemental Table S1).

### SDS-PAGE and western blot analyses

Total protein was extracted from tissue culture leaves after transient transformation using a protein extraction buffer containing 150 mM Phosphate Buffer, 10 mM EDTA, 25 mM Sodium Metabisulphite, 100 mg/L PVP, 0.1% Triton X-100, 0.1% SDS and 0.07% β-mercaptoethanol (Côté et al., 2003). Briefly, 1 ml extraction buffer was added to 50 mg ground tissues and held on ice for 10 min. All of samples were centrifuged at 13,000 rpm for 10 min at 4 °C. 800 μl of supernatant was removed to a fresh 1.5 ml tube and kept on ice for immediate use or snap frozen in liquid nitrogen and stored at − 80 °C. The total proteins were separated by 10% SDS-PAGE and stained by Coomassie Blue. The separated proteins were transferred onto a PVDF membrane as previously described^28^ and detected by Mouse anti GFP-Tag monoclonal antibody (ABclonal, USA) and HRP Goat Anti-Mouse IgG (H + L) as the secondary antibody (ABclonal, USA). SuperSignal™ West Pico PLUS Chemiluminescent Substrate (Thermo Fisher, USA) was used to detect HRP. Finally, chemiluminescence was captured by ChemiDoc MP Imaging System (Bio-Rad, USA) and analyzed by ImageJ.

### Chromatin Immunoprecipitation sequencing (ChIP-seq) assays

After transfection and successful validation of GFP signal, 8 g of leaf tissue was harvested and immediately cross-linked with 1% formaldehyde under vacuum for 15 min at 25 °C. The cross-linking was stopped by adding glycine to the final concentration of 0.125 M for 5 min under vacuum. The cross-linked samples were washed three times with double distilled water. The ChIP assay based on GFP Monoclonal Antibody (Invitrogen, USA) was performed as described previously^29,30^. Chromatin samples without GFP antibody immunoprecipitation were used as the control. DNA was purified and the library was prepared by NEBNext Ultra II DNA Library Prep Kit (New England Biolabs, USA). The DNA libraries were sequenced by Illumina NextSeq 2000 next-generation sequencing system from SUNYMAC (SUNY Molecular Analysis Core).

For ChIP-Seq data processing, the paired-end reads were trimmed by Trimmomatic^31^. After trimming the adapter sequence, clean reads were mapped to the *Populus tremula* × *alba* genome (https://urgi.versailles.inra.fr/Species/Forest-trees/Populus/Clone-INRA-717-1B4) by Bowtie2 with default parameters^32^. Mapped reads with low mapping quality (MAPQ < 30) or multiple duplicates removed by SAMtools^33^. Enriched peaks were identified by MACS^34^. We defined the region of a target gene as the range from 2.5 kb upstream of transcription start site (TSS) to transcription terminal site (TTS). The target genes of each peak were annotated by annotate Peak function in Chipseeker R package^35^. plotProfile and plotHeatmap in deepTools were used for peak visualization^36^. To identify DNA motifs, the peak files were searched for enriched DNA motifs using the MEME^37^. Peak files were visualized by Integrated Genome Browser^38^.

## Results

### TFs bind to the promoter of PtrPARVUS2

**Figure 1:**
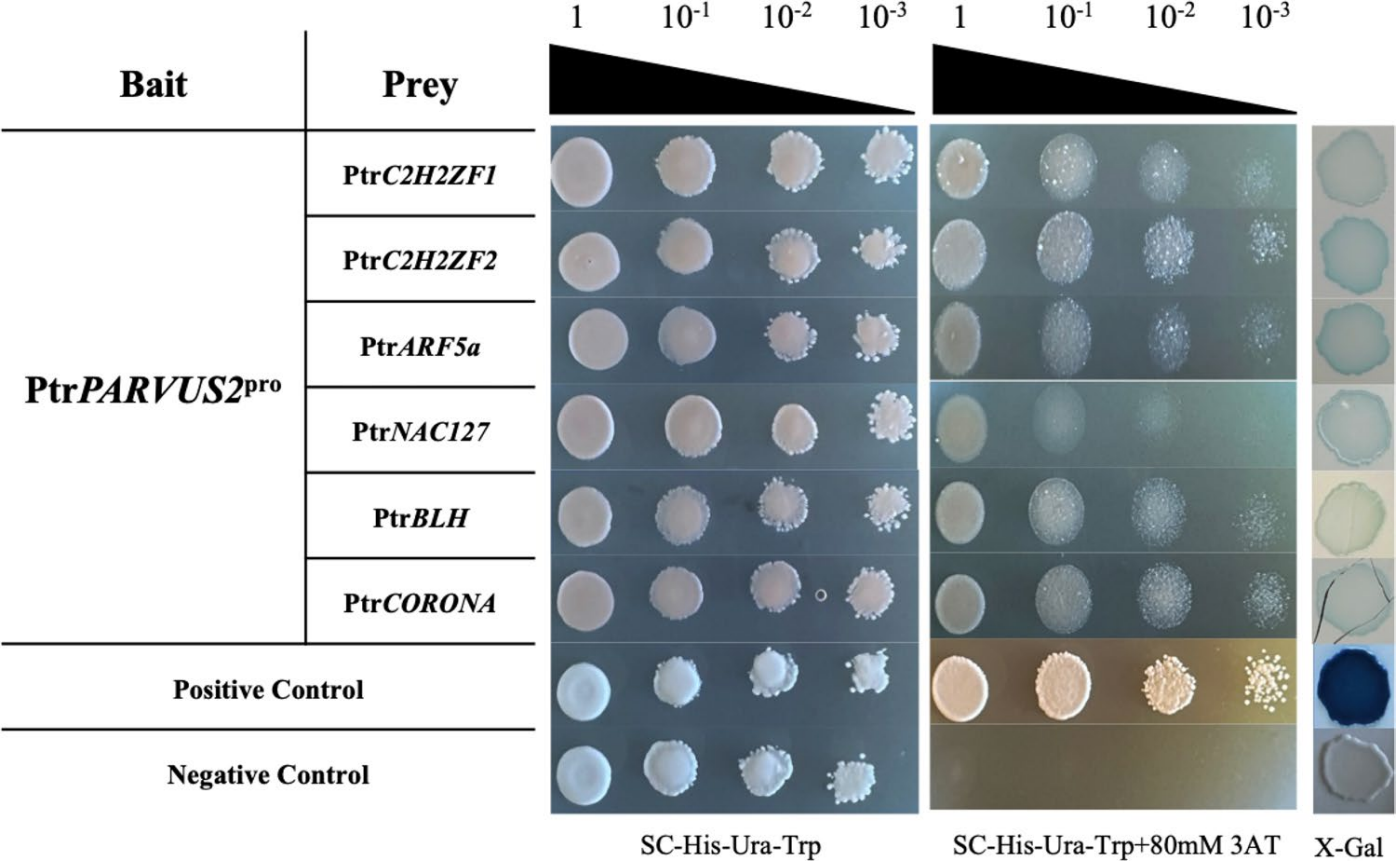
Y1H analysis of interactions between six transcription factors and the Ptr*PARVUS2* promoter. pMW#2-Ptr*PARVUS2*^pro^ and pMW#3-Ptr*PARVUS2*^pro^ with *HIS3* and *LacZ* reporter genes were used for the bait, six TFs fused with activation domain were used as prey. The empty pDEST-AD vector was used as the negative control, the positive yeast stain was a gift from Sarah Hall (Syracuse University).

To determine whether the 42 TFs from the xylem biased library interact with the promoter of Ptr*PARVUS2*, the 2.1 kb promoter was cloned in pMW#2 and pMW#3 vectors, which harbor *HIS3* and *LacZ* report genes for Y1H assays. Of the transformed yeast cells with two different reporters, only Ptr*C2H2ZF1*, Ptr*C2H2ZF2*, Ptr*ARF5a*, Ptr*BLH*, Ptr*NAC127* and Ptr*CORONA* grew on SC–His/–Ura/–Trp medium with 0 mM and 80 mM 3-AT (Fig. 1, Supplemental Fig. S1). At the same time, X-Gal was active and turned blue in these transformed yeast cells. These results suggested that Ptr*C2H2ZF1*, Ptr*C2H2ZF2*, Ptr*ARF5a*, Ptr*NAC127*, Ptr*BLH* and Ptr*CORONA* can bind to the 2.1 kb promoter of Ptr*PARVUS2*.

### TFs localize to the nucleus

**Figure 2:**
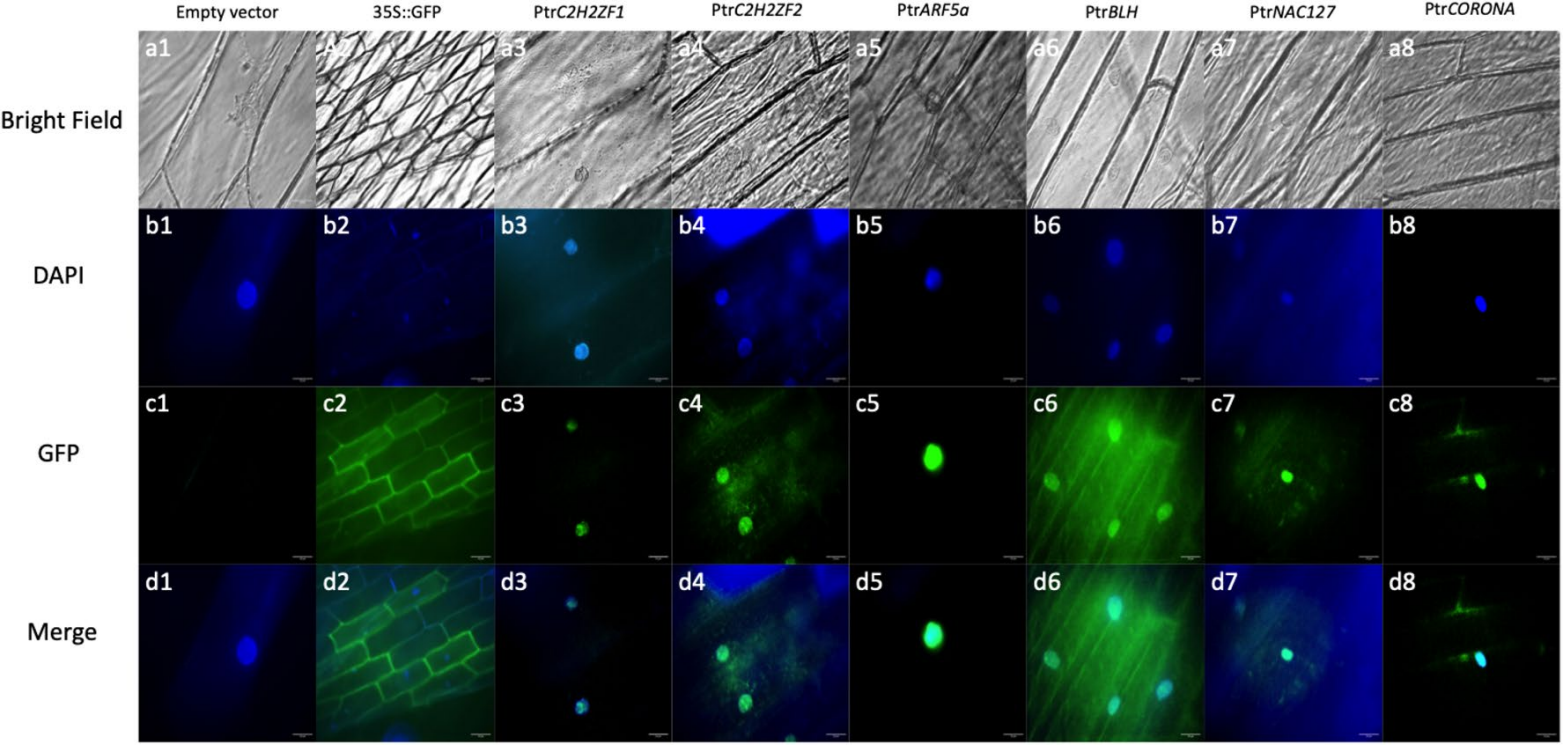
Subcellular localization of Ptr*C2H2ZF1*, Ptr*C2H2ZF2*, Ptr*ARF5a*, Ptr*NAC127*, Ptr*BLH* and Ptr*CORONA*. Subcellular localization was performed in onion epidermis by *Agrobacterium* infection. Bright field (**a1**–**a8**), DAPI (**b1**–**b8**) and GFP (**c1**–**c8**) were observed captured by Nikon SMZ1500 microscope with an Endow GFP Longpass filter (excitation: 460–500 nm and emission: 500 nm) and a TRITC (DsRed) filter (excitation: 530–560 nm and emission: 590–650 nm). Merged images (**d1**–**d8**) were analyzed by Image. The empty vector and 35S::GFP were used as the negative and positive controls, DAPI staining was used for labeling nuclei.

To investigate the subcellular localization of six TFs in *vivo*, we transformed CaMV35S::TF-GFP into onion epidermal cells by *Agrobacterium*-mediated transient transformation, using CaMV35S empty vector and CaMV35S::GFP as negative and positive controls. CaMV35S::Ptr*C2H2ZF1*-GFP, CaMV35S::Ptr*C2H2ZF2*-GFP, CaMV35S::Ptr*ARF5a*-GFP, CaMV35S::Ptr*NAC127*-GFP and CaMV35S::Ptr*CORONA*-GFP expression was observed in the nuclei, which were also stained by DAPI (Fig. 2). CaMV35S::Ptr*BLH*-GFP was expressed in both the cytoplasm and nucleus. By contrast, CaMV35S::GFP signals were uniformly distributed throughout the cell, and there was no GFP signal in empty vector transformed cell, indicating that Ptr*C2H2ZF1*, Ptr*C2H2ZF2*, Ptr*ARF5a*, Ptr*NAC127* and Ptr*CORONA* are nuclear proteins. The Ptr*BLH* expression pattern indicated that it might have another function besides as a transcription factor.

### TFs function as transcriptional repressors of PtrPARVUS2

**Figure 3:**
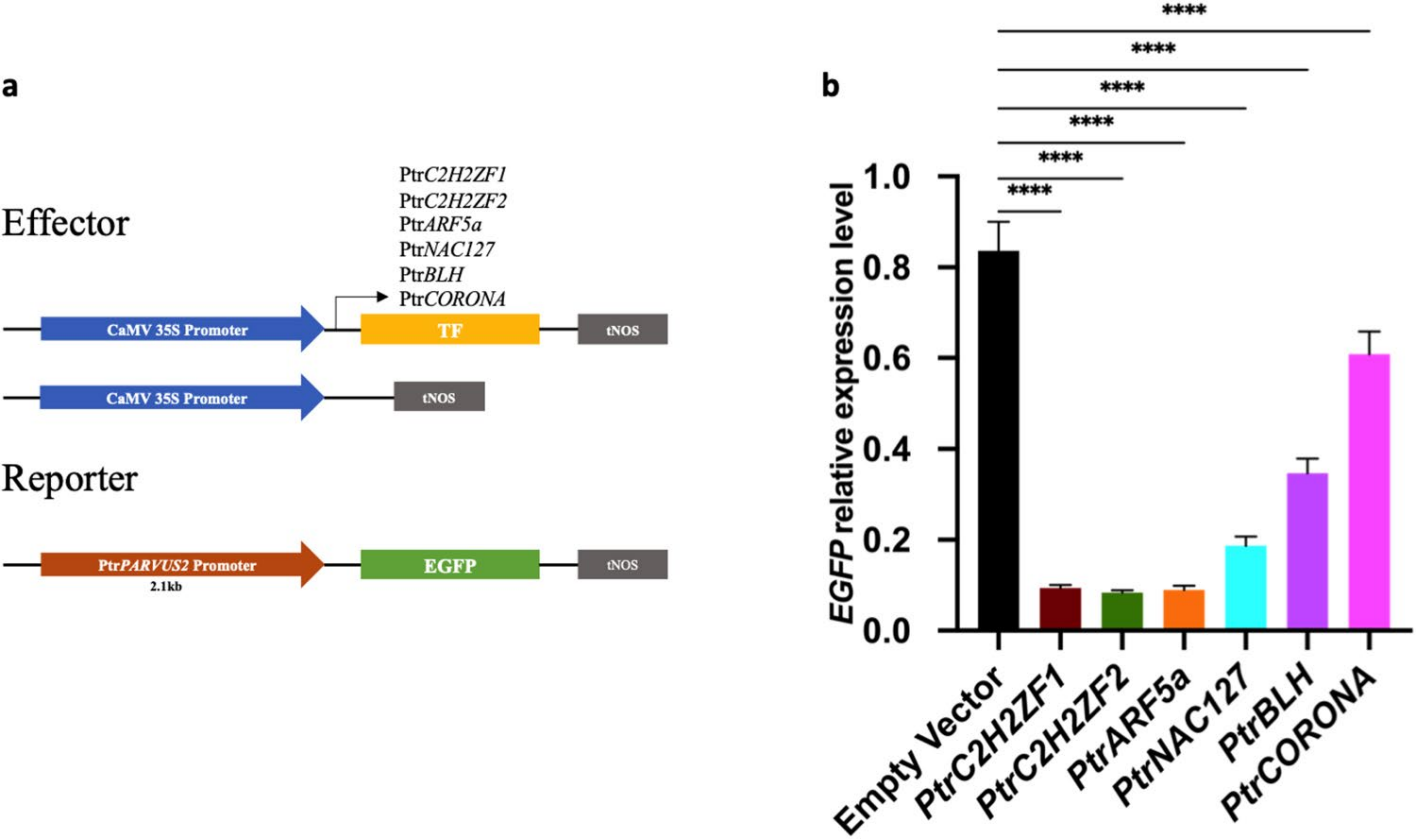
(**a**) Diagrams of the effector and reporter vectors used in transient expression assays. (**b**) Analysis of EGFP in leaf tissues after transient expression by RT-qPCR. Expression values are average of three biological replicates ± standard error of the mean (SEM), normalized to the reference UBQ11 and EF1β, respectively. The error bars represent standard deviations from three biological replicates. ****P < 0.0001 based on *t*-test.

Previous transcriptome analysis showed that PtrBLH and PtrNAC127 had the opposite expression pattern in the stem as PtrPARVUS2, which had expression in 1st-4th stem internodes, but not in 7th-8th stem internodes^15^ indicated that PtrBLH and PtrNAC127 could be repressors of PtrPARVUS2. To gain insight into the transcriptional role of these six TFs, the TFs were transiently expressed in leaf tissues of PtrPARVUS2^pro^::EGFP transgenics through agroinfiltration (Fig. 3a). EGFP transcriptional levels were detected by RT-qPCR, using empty vector as the control. The results show that EGFP transcriptional levels were decreased after overexpressing Ptr*C2H2ZF1*, Ptr*C2H2ZF2*, Ptr*ARF5a*, Ptr*NAC127*, Ptr*BLH* and Ptr*CORONA* (Fig. 3b), indicating that these six TFs can negatively regulate Ptr*PARVUS2*.

### TFs target genes identified by ChIP-Seq

**Figure 4:**
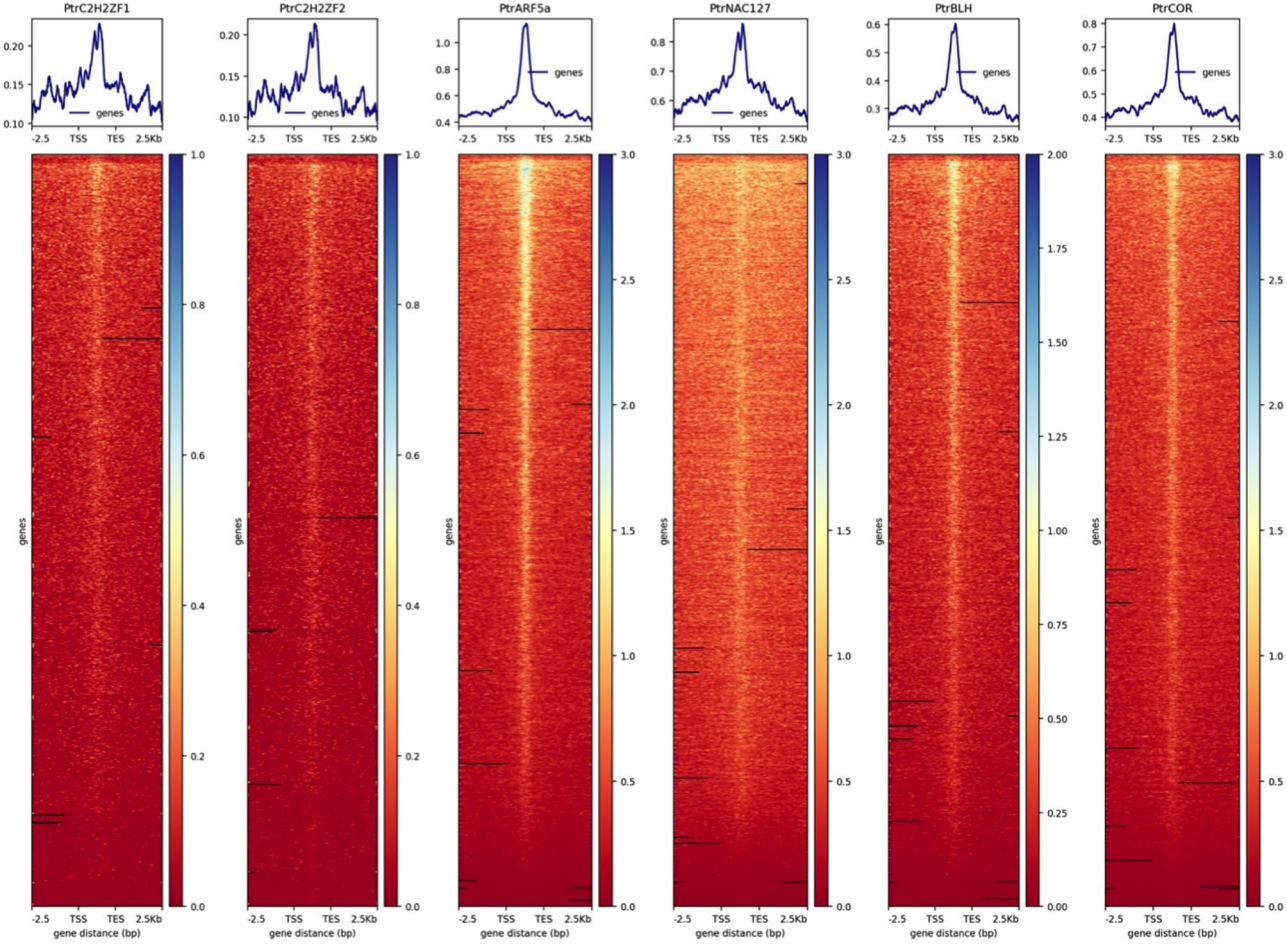
The distribution of ChIP signals in the gene body and flanking regions. TSS, transcription start site; TTS, transcription termination site; 2.5, 2.5 kb downstream of the TTS; -2.5, 2.5 kb upstream of the TSS.

**Figure 5:**
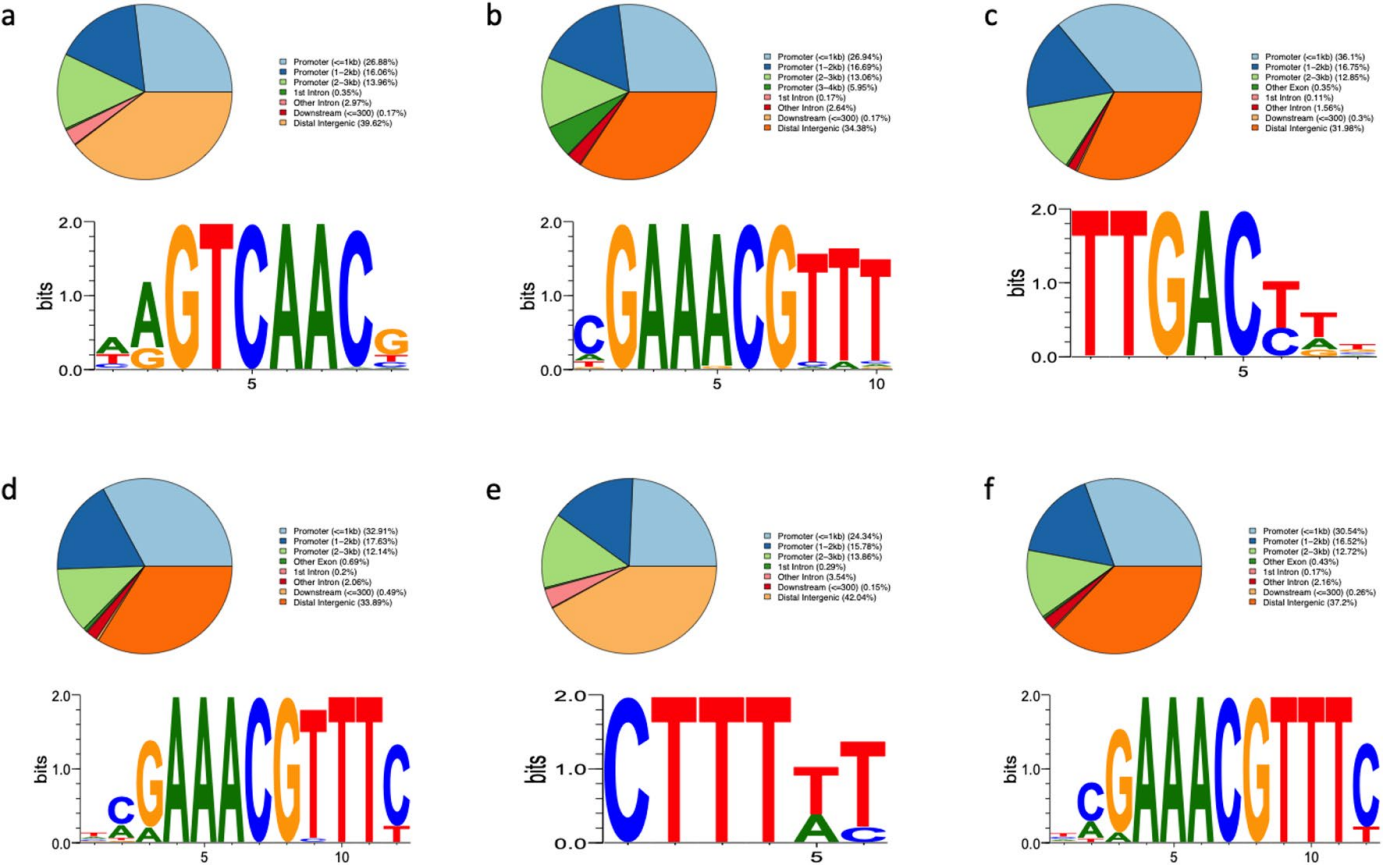
Distribution of peaks in different genomic regions and correspondent motifs. In each pattern from a to f, pie chart showing the distribution of ChIP peaks in genic and distal intergenic regions in each TF, the motif showed as sequence LOGO was significantly enriched in TF-binding regions in each TF.

To identify what additional genes can be targeted by these six TFs, ChIP-Seq was performed by immunoprecipitating TF-GFP fusion protein and the cross-linked DNA. ~ 15 million raw reads per sample were obtained from sequencing. Ptr*C2H2ZF1*, Ptr*C2H2ZF2*, Ptr*ARF5a*, Ptr*BLH*, Ptr*NAC127* and Ptr*CORONA* bind predominantly to promoter regions, particularly the regions around the transcriptional start site (TSS) and that have a moderate enrichment at transcribed gene body regions (Fig. 4). A total of 575 putative binding sites were identified in the Ptr*C2H2ZF1* sample, with the majority (54%) located in the promoter, 42% in distal intergenic regions, and 4% in other areas such as introns and exons (Fig. 5a). Similarly, in the Ptr*C2H2ZF2* sample, a total of 607 putative binding sites were found, with 60% in the promoter, 37% in distal intergenic regions, and 3% in other regions (Fig. 5b). In the Ptr*ARF5a* sample, 5397 putative binding sites were identified, with 57% in the promoter, 34% in distal intergenic regions, and 9% in other regions (Fig. 5c). In the *PtrBLH* sample, 1022 putative binding sites were identified, with 66% in the promoter, 32% in distal intergenic regions, and 2% in other regions (Fig. 5d). For Ptr*NAC127*, a total of 682 putative binding sites were found, with 63% in the promoter, 34% in distal intergenic regions, and 3% in other regions (Fig. 5e). Lastly, in the Ptr*CORONA* sample, 1106 putative binding sites were found, with 57% in the promoter, 40% in distal intergenic regions, and 3% in other regions like introns and exons (Fig. 5f). Approximately 75% of the binding sites within the promoter regions targeted by these six TFs were concentrated within the 2 kb region, signifying their active involvement in the transcription binding process. The full list of the genes targeted by the six TFs is in Supplemental Table S3–S8.

### Binding motif analysis reveals *cis*-regulatory elements for these six TFs

To explore the binding motifs from Ptr*C2H2ZF1*, Ptr*C2H2ZF2*, Ptr*ARF5a*, Ptr*BLH*, Ptr*NAC127* and Ptr*CORONA*, 2 kb flanking sequences around all peaks were analyzed by the motif discovery tool MEME described in Methods section. The highest score motifs from each TFs were showed in Fig. 5a–f, the motif HRGTCAACB (E-value = 4.4E − 22) demonstrated statistical significance as the predominant binding motif in Ptr*C2H2ZF1* binding sites, whereas the motif GAARCGHW (E-value = 4.6E − 21) took precedence in Ptr*C2H2ZF2* binding sites. Furthermore, among Ptr*ARF5a* and *PtrBLH* binding sites, TTGACYD (E-value = 4.3E − 340) and RAAACGYTTY (E-value = 4.60E − 48) emerged as the most statistically relevant. Likewise, the motif CTTTWY (E-value = 4.70E − 27) displayed notable significance in Ptr*NAC127* binding sites, while the motif HRAAACGTTTY (E-value = 4.80E − 52) was statistically relevant in Ptr*CORONA* binding sites. All the highest score motifs listed above are present in the PtrPARVUS2 2 kb-promoter region (Supplemental Fig. S2). These results indicate that the genes with these motifs in promoter regions could be regulated by these six TFs directly.

### Regulatory network among six TFs

**Figure 6:**
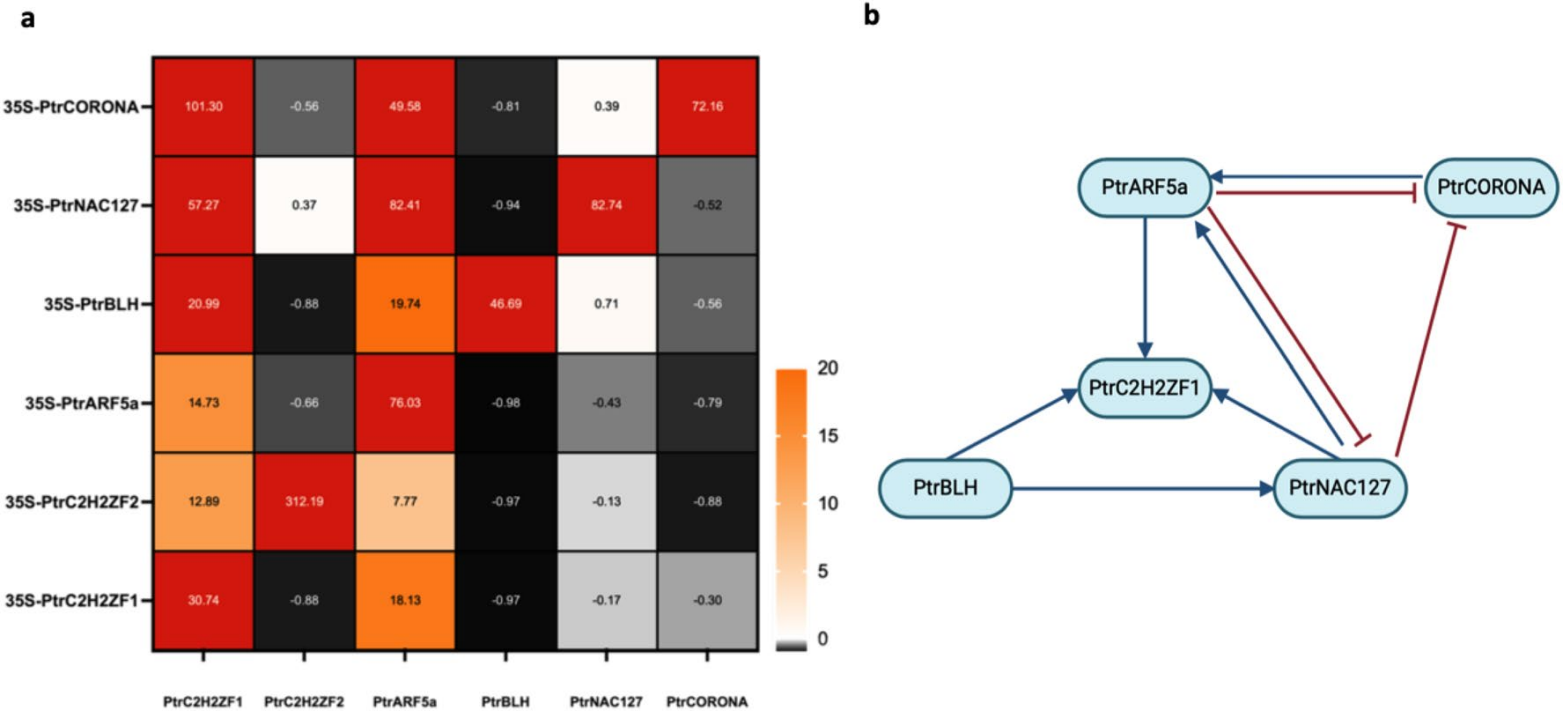
A proposed regulatory network among multiple TFs. (**a**) RT-qPCR was performed after transient expression of six TFs. The y-axis illustrated the overexpressed 6 TFs, while the x-axis depicted the detected expression levels of 6 TFs. Expression values are the average of three biological replicates ± standard error of the mean (SEM), normalized to the reference UBQ11 and EF1β, respectively. The scale bar showed the range of relative expression levels. (**b**) Model of regulation network among 6 TFs in poplar based on RT-qPCR and ChIP-seq assay.

To shed light on the regulatory network in this group of SCW-related TFs, Ptr*C2H2ZF1*, Pt*rC2H2ZF2*, Ptr*ARF5a*, Ptr*NAC127*, Ptr*BLH*, and Ptr*CORONA* were transiently expressed in 717-1B4 leaf tissue. RT-qPCR and ChIP-seq were applied to determine their putative regulatory relationships. Ptr*C2H2ZF1* and Ptr*ARF5a* functioned as the repressors for Pt*rC2H2ZF2*, Ptr*NAC127*, Ptr*BLH*, and Ptr*CORONA* expression (Fig. 6a). The integration of RT-qPCR results and ChIP-seq assays revealed two negative feedback loops (Fig. 6b). Ptr*ARF5a* can inhibit the expression of Ptr*NAC127* and Ptr*CORONA*, while Ptr*NAC127* and Ptr*CORONA* had the ability to activate Ptr*ARF5a* expression. Additionally, Ptr*ARF5a* served as an activator for Ptr*C2H2ZF1* transcription and Ptr*C2H2ZF1* was identified as a downstream target for Ptr*ARF5a*, Ptr*NAC127*, and Ptr*BLH*.

GO enrichment analysis and promoter phylogenetic analysis indicate the potential functions of six TFs.

ChIP-seq analysis indicated that Ptr*C2H2ZF1*, Ptr*C2H2ZF2*, Ptr*ARF5a*, Ptr*BLH*, Ptr*NAC127* and Ptr*CORONA* can bind to the promoter region of Ptr*PARVUS2* (Supplemental Fig. S2). Among the target genes of Ptr*ARF5a* are, Ptr*GT8E* (Potri.007G031700), Ptr*PARVUS14* (Potri.014G040300), g*lucuronic acid substitution of xylan 1A* (Ptr*GUX1A*) (Potri.007G107200), *endo-1,4-β-glucanase 9A1* (Ptr*Cel9A1*) (Potri.003G151700) and *cellulose synthase 8B* (Ptr*CesA8B*) (Potri.004G059600) (Supplemental Table S5).

GO analysis shows that many of the binding sites occur in promoters of genes that are associated with uridine diphosphate glycosyltransferase (UGT) activity (Supplemental Fig. S3). When comparing UGT genes targeted by these six TFs, eight putative UGTs (Potri.004G069800, Potri.006G022500, Potri.009G095400, Potri.009G095500, Potri.014G088400, Potri.015G027700, Potri.016G017300 and Potri.017G052400) are identified in all TF samples (Supplemental Table S9). Phylogenetic analysis was performed in 2 kb promoter regions of these eight putative UGT genes, which can be classified in three different clades (Supplemental Fig. S4). Potri.015G027700 is in clade I, Potri.009G095500 and Potri.014G088400 are classified in clade II, and the remaining five putative UGTs are in clade III.

## Discussion

The present research aimed to identify TFs from a xylem biased library for their ability to regulate Ptr*PARVUS2* expression by targeting specific *cis-elements* in the promoter region. The investigation into the regulatory network controlling SCW development in plants, with a focus on the TF-mediated regulation of the Ptr*PARVUS2* gene, provides valuable insights into the molecular mechanisms underlying cell wall formation. We have showed that six TFs can bind to Ptr*PARVUS2* promoter region to activate the downstream *HIS3* and *LacZ* reporter genes in Y1H assays, and that these six TFs are negative transcriptional regulators of Ptr*PARVUS2*. These six TFs from five different families were reported to have multiple functions in cell development and different stress responses, but the function in SCW biosynthesis has not been fully studied.

Ptr*C2H2ZF1* (Potri.010G209400) and PtrC2H2ZF2 (Potri.014G066200) are from C2H2 zinc finger (C2H2-ZF) TF family. The first discovery of zinc-binding domains occurred in the protein TFIIIA from *Xenopus oocytes*. These domains play a crucial role in binding to IIA and DNA, utilizing cysteines and histidines within the enriched folding domain centered around a zinc ion^39^. The majority of classical Cys2His2 (C2H2) zinc fingers function as transcription factors, contributing to DNA binding and transcriptional regulation through the β-β-α framework of a C2H2 zinc finger module (Supplemental Fig. S5)^40^. The functions of C2H2 zinc fingers in growth regulation, stress response and epigenetic have been reported in plants^41–43^. However, the role of C2H2 zinc fingers in the SCW formation in poplar remains poorly understood.

Ptr*ARF5a* (Potri.002G024700) is from Auxin Response Factor family. 39 ARF genes have been identified in *P. trichocarpa*^44^. Each ARF protein features a conserved DNA-binding domain (DBD) at its N-terminus, followed by a middle region (MR) and a C-terminal PB1 domain^45^. The N-terminal DBD is highly conserved among ARFs, however, the MR exhibits variability depending on whether it is associated with activating or repressing functions. When the middle region (MR) is characterized by an abundance of glutamines, it functions as an activator. Conversely, a middle region rich in proline, serine, and threonine imparts repressor activity^44^. But the mechanism of activation and repression is still waiting to be elucidated. The function of ARFs in the cell wall is being revealed, as ARFs can regulate the cell elongation, targeting genes involved in cellulose and pectin biosynthesis in the primary cell wall^46^. Following treatment with IAA (Indole-3-Acetic Acid) in *Arabidopsis*, At*PARVUS* (AT1G19300), the ortholog of Ptr*PARVUS2*, exhibits upregulation within 120 minutes^47^. It indicated that auxin-related receptors could be involved in Ptr*PARVUS2* transcriptional regulation.

Ptr*BLH* (Potri.010G197300) is BEL1-Like Homeodomain protein from homeobox protein transcription factor family. Three-amino-acid-loop-extensions and a conserved proline-tyrosine-proline amino acid sequence within the homeodomain are two key characteristics of BLH protein^48^. The domains located at N-terminus and C-terminus are conserved, homeodomain serves as DNA-binding domain at its N-terminus, POX domain is conserved at C-terminus, involving in homodimer or heterodimer formation^49,50^. Eighteen *BLH* genes have been identified in the poplar genome^51^, but the role of each of these genes has not yet been elucidated. Ptr*BLH6a* has been identified as a negative regulator of genes involved in the lignin biosynthesis pathway, the overexpression mutants did not exhibit any alterations in lignin composition (S/G ratio) and content^52^. In contrast, *blh6* mutants in cotton and camellia show a reduction in lignin content^53,54^. In addition to a role in regulating SCW formation, *BLHs* are also reported to have vital roles in salt stress, heat stress and other pathogen infections^51,55^.

Ptr*NAC127* (Potri.018G068700) is from NAM/ATAF1/CUC2 (NAC) transcription factor family, the largest plant-specific TF family involved in biotic and abiotic stress response and plant development^56–58^. The NAC proteins encompass a C-terminal transcription regulation region (TRR) that is highly variable, featuring a substantial content of low-complexity amino acid repeats, and N-terminal with a 150-amino-acid DNA-binding domain (DBD) is conserved in NAC TFs^59^. The TRR acts as both activator and/or repressor to downstream target genes together with the NAC domain^60^. The NAC domain is present in all NAC proteins, with the most conserved consensus sequences being D-D/E-L-I/V, E-W-Y-F-F, G-Y-W-K, and M-H-E-Y^61^. Nuclear localization signals (NLSs) within the NAC domain have been identified, indicating their role in DNA-binding functions^62^. Overexpression of Ptr*WND1B*, Ptr*WND2B* and Ptr*WND6B*, other members of Wood-associated NAC Domain (WND) transcription factor family, results in the increased expression of IRX8, IRX9, FRA8 and GT43B involved in xylan biosynthesis in hemicellulose^63–65^. Ptr*WND4A* and Ptr*WND4B*, orthologs of At*VND4/5* with the same the NAC domain as Ptr*NAC127* in poplar, have different expression patterns; Ptr*WND4A* cannot be expressed in the petiole, and Ptr*WND4B* has an extremely low expression in the leaf^66^. When At*SND2* was overexpressed in *Arabidopsis*, xylose content did not show a significant change, but Ptr*NAC154* which is the ortholog of At*SND2* in poplar can decrease xylose content significantly after overexpression in poplar^67,68^. The ortholog of Ptr*NAC127* in Arabidopsis is At*SND4*, which can bind to the 19-bp consensus sequence named SNBEs (secondary wall NAC binding elements) to regulate downstream TFs involved in SCW formation^69,70^, and the evidence that Ptr*NAC127* can regulate the SCW biosynthesis genes was provided in this study.

Ptr*CORONA* (Potri.001G188800) is a member of Class III Homeodomain–Leucine Zipper (HD ZIP III) transcription factor family. This family is grouped in three clades: *REVOLUTA* (*REV*), *PHABULOSA/PHAVOLUTA* (*PHB/PHV*), *CORONA/AtHB15* (*CNA*)^71^. HD-ZIP III proteins contain a unique START domain located at N-terminal for steroid binding and a C-terminal MEKHLA (Met-Glu-Lys-His-Leu-Ala) domain, which is responsive to various chemical and physical stimuli^72^. The HD ZIP III genes play a crucial role in governing plant development, including meristem development, leaf polarity, and vascular development in both stems and roots^73–77^. The recent studies indicate that post-transcriptional regulation of HD ZIP III genes is associated with various abiotic stresses in a number of species^78–81^. In poplar, Ptr*REV* and Ptr*CORONA* have been reported to regulate vascular cambium development and cell differentiation during secondary growth^73,82^.

Furthermore, hierarchical regulatory networks have been previously reported for transcription factors involved in plant stress responses and secondary metabolism^83,84^. The results of ChIP-seq revealed regulatory networks among six TFs implicated in SCW biosynthesis (by their xylem-enhanced expression), including Ptr*C2H2ZF1*, Ptr*C2H2ZF2*, Ptr*ARF5a*, Ptr*NAC127*, Ptr*BLH*, and Ptr*CORONA*. Based on this data, these TFs exhibit potential hierarchical relationships and feedback loops, collectively regulating the expression of genes involved in SCW formation. Notably, Ptr*C2H2ZF1* and Ptr*ARF5a* act as repressors for Ptr*C2H2ZF2*, Ptr*NAC127*, Ptr*BLH*, and Ptr*CORONA* expression (Fig. 6), highlighting the complexity of transcriptional regulation in SCW biosynthesis.

Among the target genes of Ptr*ARF5a* are Ptr*GT8E*, Ptr*PARVUS14*, Ptr*GUX1A*, Ptr*Cel9A1* and Ptr*CesA8B*, which are key enzymes related to cell wall formation. Ptr*GT8E* and Ptr*PARVUS14* are close homologs of PtrPARVUS2 and have important roles in xylan backbone biosynthesis^85^. Ptr*GUX1A* plays a role in acetyl modification, demonstrating a proclivity for even modification along the backbone^86^. In addition to these xylan biosynthesis and modification genes, cellulose biosynthesis genes are also the targets of Ptr*ARF5a* including Ptr*Cel9A1* and Ptr*CesA8B.* Suppression of either Ptr*Cel9A1* or Ptr*CesA8B* in poplar can lead to defects in xylem cells and significant cellulose reduction in SCW^87,88^. These findings showed that Ptr*ARF5a* has a multiple regulatory function role in SCW formation, and auxin could be a key SCW formation regulator.

GO analysis reveals that numerous binding sites are located within the promoters of genes linked to UGT activity (Supplemental Fig. S3). UGTs are known to catalyze the transfer of sugar moieties from uridine diphosphate (UDP) sugar donors to various acceptor molecules, leading to the formation of glycosidic bonds^89^. In the context of SCW biosynthesis, UDP-glycosyltransferases are involved in the glycosylation of hemicelluloses like xylan, influencing its structure and function^90^. The substrate specificity of UGTs determines the precise glycosylation patterns of hemicelluloses within SCW. In poplar, 191 putative UGTs have been identified^91^, but their regulatory mechanisms have not been elucidated yet. The eight putative UGTs regulated by all six TFs can be grouped into three clades using promoter phylogenetic analysis (Supplemental Fig. S4). According to previous research, genes in clades I and III are associated with two different environmental stresses. Potri.015G027700, classified in clade I, has been significantly downregulated under heat shock in poplar^92^ and Potri.006G022500 and Potri.017G052400 from clade III are activated under drought stress^91,93, 94^. Understanding the regulation of UGTs in different plant species can provide valuable insights into the variability of cell wall structures and properties. The intricate network of TFs involved in SCW biosynthesis interacts with UGTs to coordinate the expression of genes responsible for hemicellulose synthesis. This interplay ensures the precise spatiotemporal regulation of cell wall components. In this study, six different TFs have shown the abilities to regulate PtrPARVUS2 from GT8 family through Y1H, ChIP-seq and transient expression (Figs. 1, 3 and 5). The identification of TFs associated with UGTs will contribute to a more comprehensive view of the regulatory mechanisms governing SCW formation.

## Conclusion

Our results show that Ptr*C2H2ZF1*, Ptr*C2H2ZF2*, Ptr*ARF5a*, Ptr*NAC127*, Ptr*BLH* and Ptr*CORONA* can bind to PtrPARVUS2 promoter in vivo and, apart from Ptr*BLH* are localized in the nuclei. These six TFs suppress Ptr*PARVUS2* transcription by targeting different *cis-elements* in the promoter region directly or indirectly. These results provide valuable insights into the transcriptional regulatory mechanisms of Ptr*PARVUS2* in plant development. Further understanding the transcriptional network regulating Ptr*PARVUS2* will lead to novel strategies for engineering xylan in hemicellulose.

### Supplementary Information

Supplementary Information 1.

Supplementary Information 2.

## Data Availability

The data underlying this article are available in the article and in its online Supplemental material. The ChIP-Seq datasets generated and analyzed during the current study are available in the GEO repository (GSE263488).
