# Supplementary material for: The transcriptional regulation of a putative hemicellulose gene, PtrPARVUS2 in poplar
Source: Sci Rep. 2024 Jun 1;14:12592. doi: 10.1038/s41598-024-63408-x (PMC11144201; doi:10.1038/s41598-024-63408-x)
Supplement: Supplementary file 1 — Supplementary Information 1. [file 41598_2024_63408_MOESM1_ESM.docx]

**Title: The transcriptional regulation of a putative hemicellulose gene, PtrPARVUS2 in poplar**

**Journal Name: Scientific Reports**

**Dan Wang^1^, Heather D. Coleman ^1*^**

^1^Department of Biology, Syracuse University, Syracuse, NY, 13244 USA

ORCID

H. D. Coleman: 0000-0002-4923-601X

D. Wang: 0000-0002-0898-0854

***Correspondence:**  
H. D. Coleman 
hcoleman@syr.edu

315-443-0453

**Supplementary Information**

Table S1. Primers used in this study.

Table S2. Plasmids used in this study.

Table S3. List of genes targeted by PtrC2H2ZF1 (Excel file).

Table S4. List of genes targeted by PtrC2H2ZF2 (Excel file).

Table S5. List of genes targeted by PtrARF5a (Excel file).

Table S6. List of genes targeted by PtrBLH (Excel file).

Table S7. List of genes targeted by PtrNAC127 (Excel file).

Table S8. List of genes targeted by PtrCORONA (Excel file).

Table S9. UTG genes identified in all samples (Excel file).

Figure S1. Negative Y1H screening results.

Figure S2. Binding site visualization in PtrPARVUS2 promoter region.

Figure S3. GO analysis were performed in ChIP-seq data from Ptr*C2H2ZF1*, Ptr*C2H2ZF2*, Ptr*ARF5a*, Ptr*BLH*, Ptr*NAC127* and Ptr*CORONA* samples.

Figure S4. Phylogenetic analysis of promoter region from 8 putative UGT genes.

Figure S5. The structures of six transcription factors were predicted by AlphaFold Protein Structure Database (<https://alphafold.ebi.ac.uk>).

**Supplemental Table S1**. Primers used in this study (*From Brunner et al., 2004).

| Primer Name | Sequence (5’ to 3’) | Description |
| --- | --- | --- |
| PARVUS-Fwd-XbaI | CCCCTGCAGGGGAGCATACTACTCGTAAAAGAGACAGCC | Forward primer to clone the promoter of Ptr*PARVUS2*, XbaI site added |
| PARVUS-Rvs-SbfI | GCTCTAGAGCTTTCATATTGGTTTTAGAAGGGGGTTTTTAG | Reverse primer to clone the promoter of Ptr*PARVUS2*, SbfI site added |
| Ptr*C2H2ZF1*-Fwd-XbaI | TCTAGAATGAAGAGAGGTCTGCACGAGAG | Forward primer to clone CDS of Ptr*C2H2ZF1*, XbaI site added |
| Ptr*C2H2ZF1*-Rvs-AscI | GGCGCGCCAAAAGAAACAATTGACCATAGGAGCTG | Reverse primer to clone CDS of Ptr*C2H2ZF1*, AscI site added |
| Ptr*C2H2ZF2*-Fwd-XbaI | TCTAGAATGGCTTTGCTTGTGGATCAAC | Forward primer to clone CDS of Ptr*C2H2ZF2*, XbaI site added |
| Ptr*C2H2ZF2*-Rvs-AscI | GGCGCGCCATAACTGGTCCGCTCTCACATCAG | Reverse primer to clone CDS of Ptr*C2H2ZF2*, AscI site added |
| Ptr*NAC127*-Fwd-XbaI | TCTAGAATGAGTACTAGGTGTAGCATGGCTTC | Forward primer to clone CDS of Ptr*NAC127*, XbaI site added |
| Ptr*NAC127*-Rvs-AscI | GGCGCGCCACCCATGATGATCCTGGTTGCC | Reverse primer to clone CDS of Ptr*NAC127*, AscI site added |
| Ptr*ARF5a*-Fwd-SpeI | ACTAGTATGGGTTCTGCTGAAGAGAAAATCAAAAC | Forward primer to clone CDS of Ptr*ARF5a*, SpeI site added |
| Ptr*ARF5a*-Rvs-AscI | GGCGCGCCAAGCATGGATACCCTCTGTGATGG | Reverse primer to clone CDS of Ptr*ARF5a*, AscI site added |
| Ptr*BLH*-Fwd-SpeI | ACTAGTATGGCACAAAACTTTGAACCCTTTCATG | Forward primer to clone CDS of Ptr*BLH*, SpeI site added |
| Ptr*BLH*-Rvs-AscI | GGCGCGCCACTCTTTCCCAAAATGCTGCTTTTCTAGTTC | Reverse primer to clone CDS of Ptr*BLH*, AscI site added |
| Ptr*CORONA*-Fwd-SpeI | ACTAGTATGATGGCAATGTCCTGCAAGGATGG | Forward primer to clone CDS of Ptr*CORONA*, SpeI site added |
| Ptr*CORONA*-Rvs-AscI | GGCGCGCCAAACAAAAGACCAGTTTATAAACATAAAGCAGATGC | Reverse primer to clone CDS of Ptr*CORONA*, AscI site added |
| UBQ11-Fwd* | GTTGATTTT GCTGGGAAGC | Forward primer for qPCR |
| UBQ11-Rvs* | GATCTTGGCCTTCACGTTGT | Reverse primer for qPCR |
| EF1β-Fwd* | GGCATTAAGTTTTGTCGGTCTG | Forward primer for qPCR |
| EF1β-Rvs* | GCGGTTCATCATTTCATCTGG | Reverse primer for qPCR |
| GFP-Fwd | CCGACCACTACCAGCAGAACAC | Forward primer for qPCR |
| GFP-Rvs | TCACGAACTCCAGCAGGACCAT | Reverse primer for qPCR |

**Supplemental Table S2**. All plasmids used in this study.

| **Recombinant DNA vector** | **Purpose** |
| --- | --- |
| pGFPGUSPlus-PtrPARVUS2^pro^::EGFP | Stable transgenic lines |
| pMW#2-PtrPARVUS2^pro^ | Bait with *HIS3* reporter gene for Y1H |
| pMW#3-PtrPARVUS2^pro^ | Bait with *LacZ* reporter gene for Y1H |
| pMDC84-CaMV35S::Ptr*C2H2ZF1* | Transient expression in PtrPARVUS2^pro^::EGFP stable transgenic lines |
| pMDC84-CaMV35S::Ptr*C2H2ZF2* | Transient expression in PtrPARVUS2^pro^::EGFP stable transgenic lines |
| pMDC84-CaMV35S::Ptr*ARF5a* | Transient expression in PtrPARVUS2^pro^::EGFP stable transgenic lines |
| pMDC84-CaMV35S::Ptr*BLH* | Transient expression in PtrPARVUS2^pro^::EGFP stable transgenic lines |
| pMDC84-CaMV35S::Ptr*NAC127* | Transient expression in PtrPARVUS2^pro^::EGFP stable transgenic lines |
| pMDC84-CaMV35S::Ptr*CORONA* | Transient expression in PtrPARVUS2^pro^::EGFP stable transgenic lines |
| pMDC84-CaMV35S::Ptr*C2H2ZF1-*GFP | Transient expression in 717 for ChIP-Seq |
| pMDC84-CaMV35S::Ptr*C2H2ZF2*::GFP | Transient expression in 717 for ChIP-Seq |
| pMDC84-CaMV35S::Ptr*ARF5a-*GFP | Transient expression in 717 for ChIP-Seq |
| pMDC84-CaMV35S::Ptr*BLH-*GFP | Transient expression in 717 for ChIP-Seq |
| pMDC84-CaMV35S::Ptr*NAC127-*GFP | Transient expression in 717 for ChIP-Seq |
| pMDC84-35S::Ptr*CORONA-*GFP | Transient expression in 717 for ChIP-Seq |


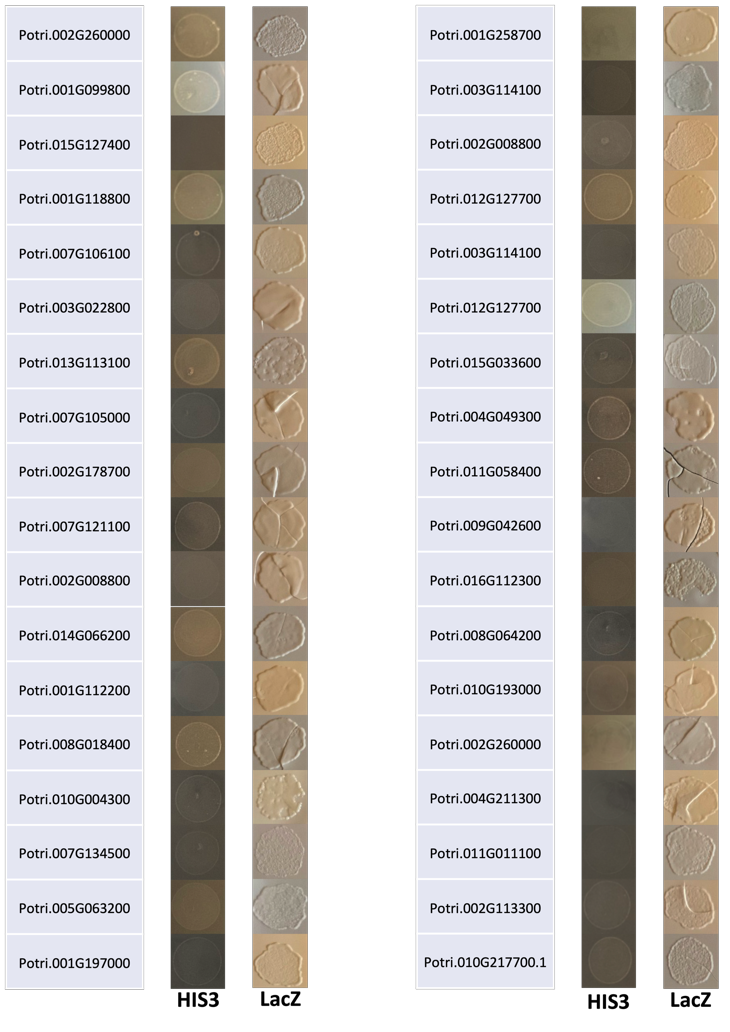


**Supplemental Figure S1. N**egative Y1H screening results. A total of 42 TFs from a xylem-enriched library were screened, 36 of the TFs were negative in both the *HIS3* and *LacZ* assays.


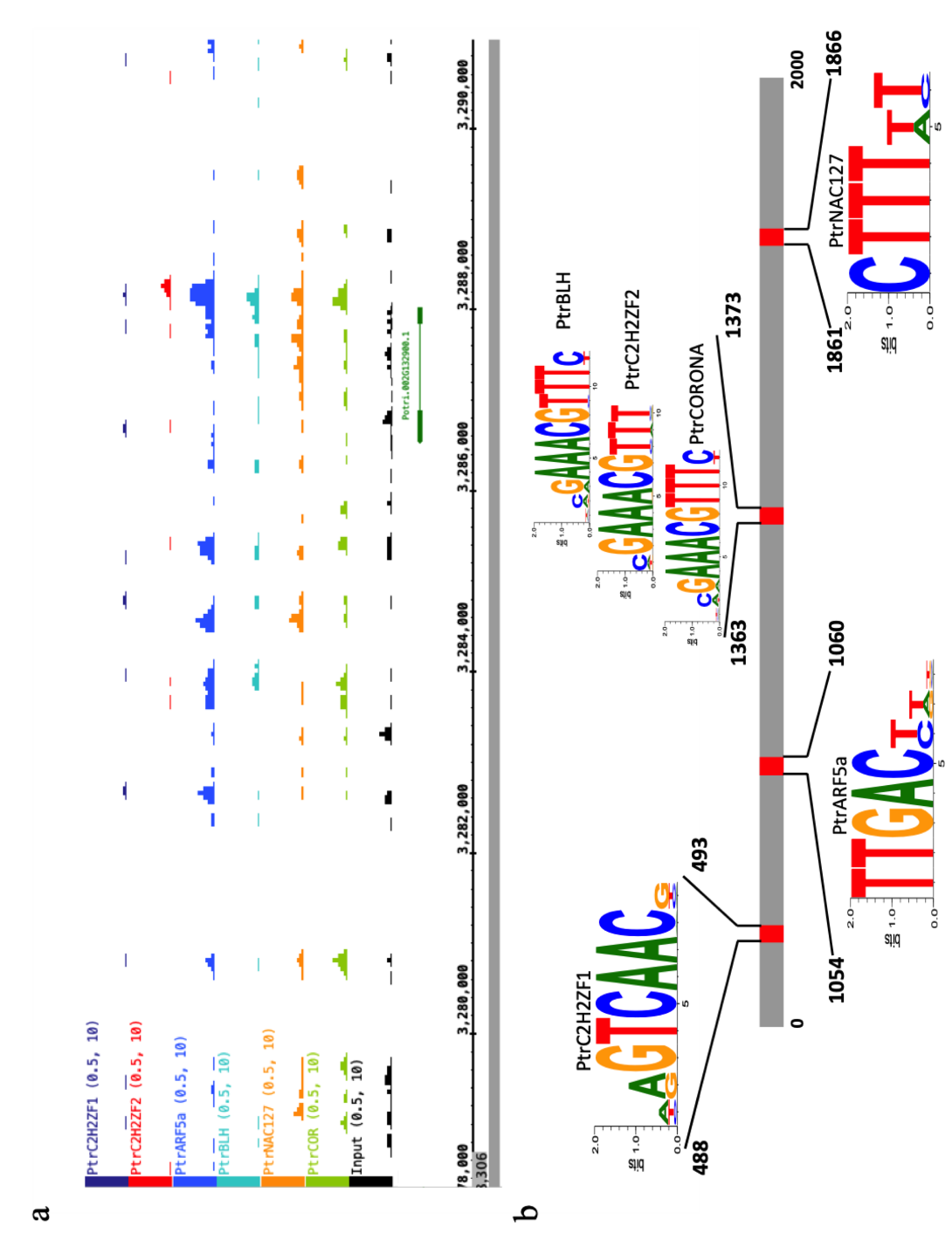


**Supplemental Figure S2.** Binding site visualization in PtrPARVUS2 promoter region. (a) Peak files from ChIP-seq were visualized by Integrated Genome Browser. Compared to the input sample, Ptr*C2H2ZF1*, Ptr*C2H2ZF2*, Ptr*ARF5a*, Ptr*BLH*, Ptr*NAC127* and Ptr*CORONA* samples have enriched peaks in the promoter region of PtrPARVUS2. (b) The details of binding sites in PtrPARVUS2 2kb-promoter region. PtrC2H2ZF1 binding site (488bp-493bp), PtrARF5a binding site (1054bp-1060bp), PtrCORONA binding site (1363bp-1373bp), PtrC2H2ZF2 binding site (1364bp-1371bp), PtrBLH binding site (1364bp-1373bp), PtrNAC127 binding site (1861bp-1866bp).

| 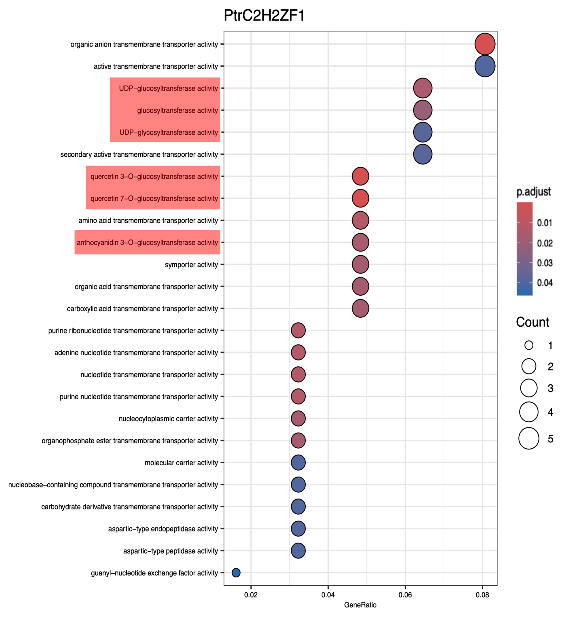 | 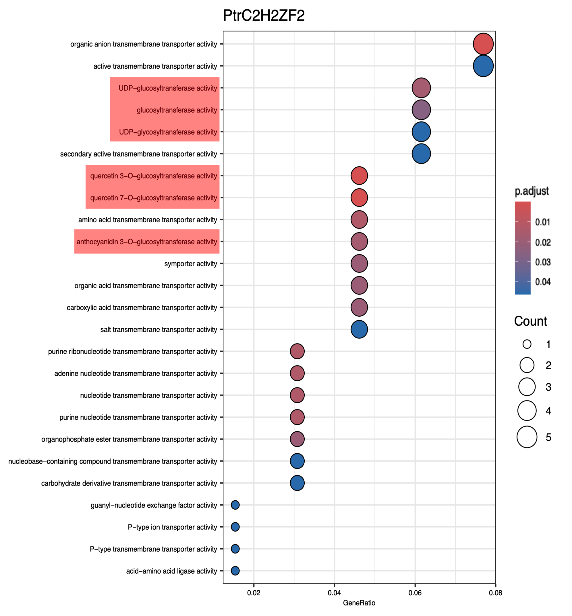 |
| --- | --- |
| 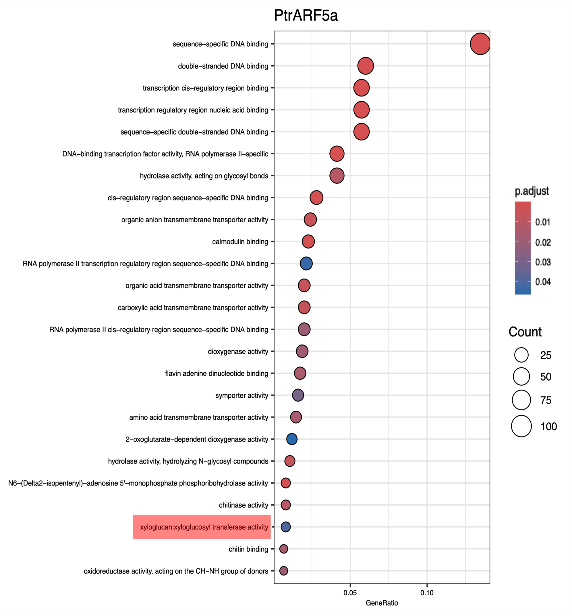 | 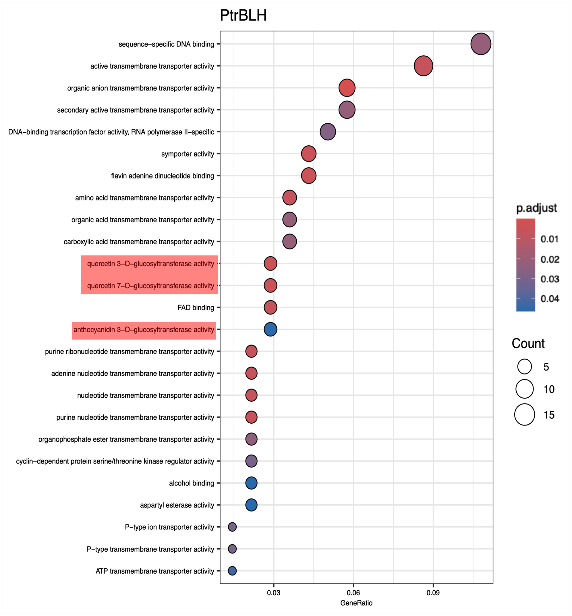 |
| 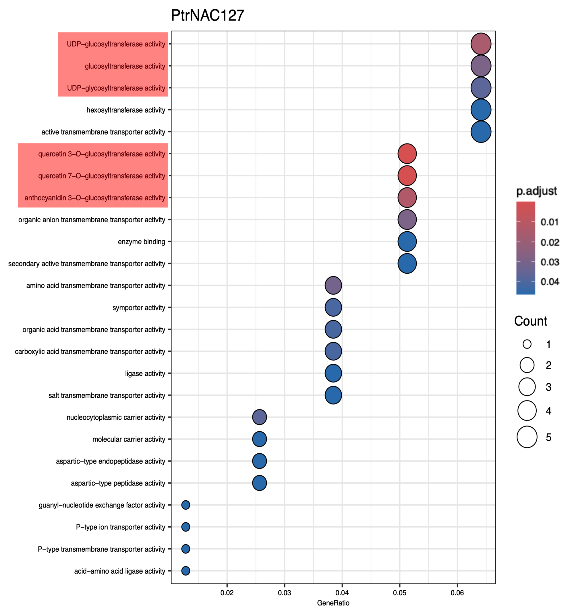 | 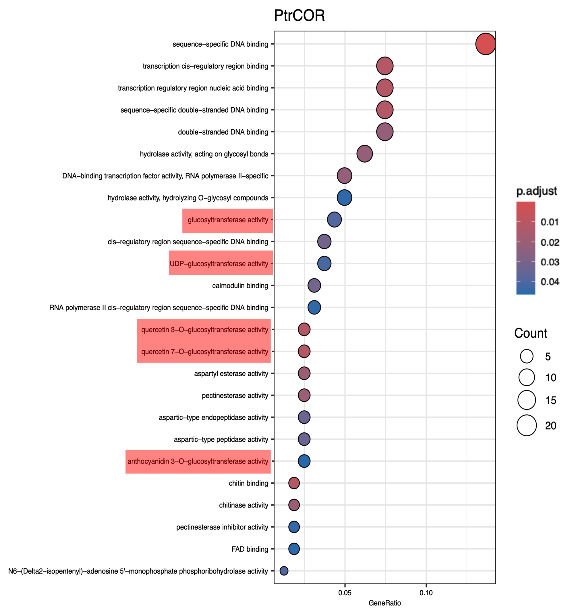 |

**Supplemental Figure S3.** GO analysis were performed in ChIP-seq data from Ptr*C2H2ZF1*, Ptr*C2H2ZF2*, Ptr*ARF5a*, Ptr*BLH*, Ptr*NAC127* and Ptr*CORONA* samples. Red squares indicate functions related to glycosyltransferase activity.

**Supplemental Figure S4.** Phylogenetic analysis of promoter region from eight putative UGT genes. Alignment and phylogeny were generated by Clustal Omega (https://www.ebi.ac.uk/jdispatcher/msa/clustalo) using 2kb upstream sequence of 5’UTR. The numbers at the nodes indicate bootstrap values in percentage of 1,000 trials.


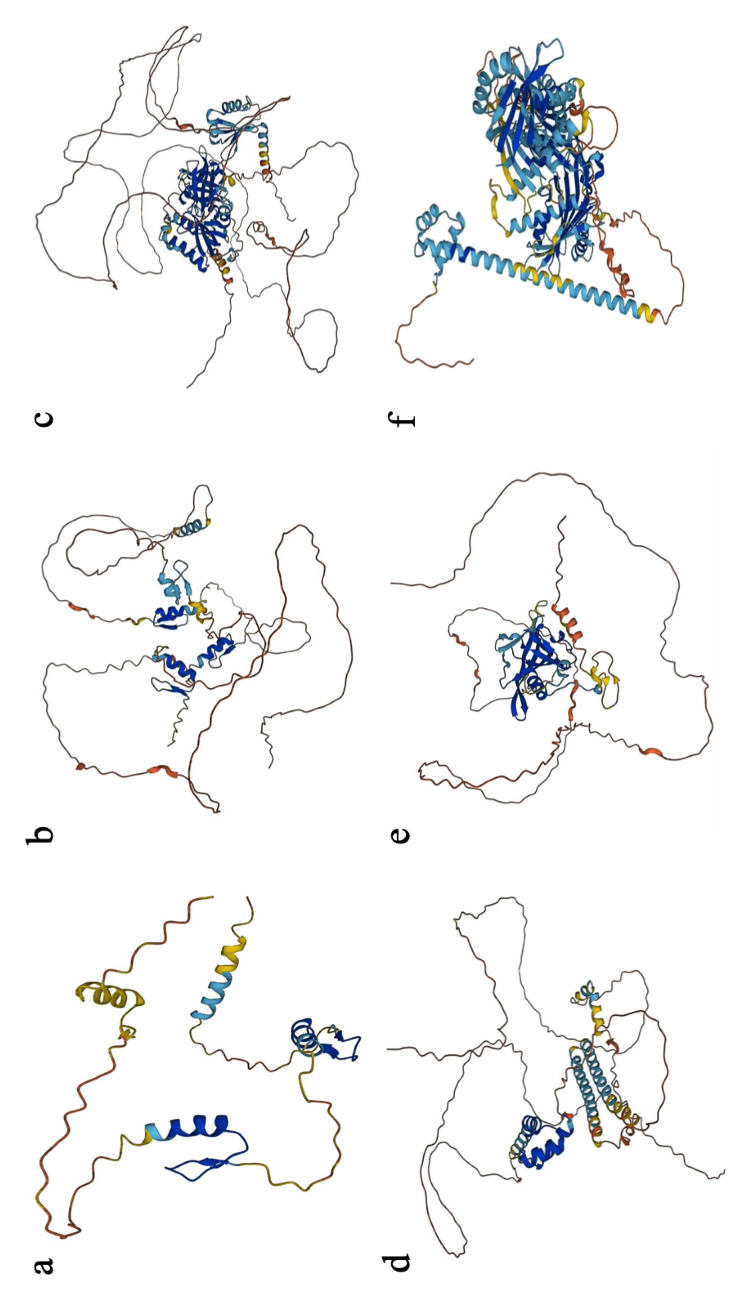


**Supplemental Figure S5.** The structures of six transcription factors were predicted by AlphaFold Protein Structure Database (<https://alphafold.ebi.ac.uk>), (a) Ptr*C2H2ZF1* (Potri.010G209400), (b) Ptr*C2H2ZF2* (Potri.014G066200), (c) Ptr*ARF5a* (Potri.002G024700), (d) Ptr*BLH* (Potri.010G197300), (e) Ptr*NAC127* (Potri.018G068700) and (f) Ptr*CORONA*(Potri.003G050100).
